# A proposed analytical framework for qualitative evaluation of access to medicines from a health systems perspective

**DOI:** 10.1186/s13104-024-06764-1

**Published:** 2024-06-07

**Authors:** I. R. Joosse, H. A. van den Ham, A. K. Mantel-Teeuwisse, F. Suleman

**Affiliations:** 1 Utrecht WHO Collaborating Centre for Pharmaceutical Policy and Regulation, Division of Pharmacoepidemiology and Clinical Pharmacology, Utrecht Institute for Pharmaceutical Sciences (UIPS), Utrecht University, Universiteitsweg 99, Utrecht, 7324, 3584 CG The Netherlands; 2 WHO Collaborating Centre for Pharmaceutical Policy and Evidence Based Practice, School of Health Sciences, University of KwaZulu-Natal, Durban, South Africa

## Abstract

**Objective:**

Despite global recognition that access to medicines is shaped by various interacting processes within a health system, a suitable analytical framework for identifying barriers and facilitators from a system’s perspective was needed. We propose a framework specifically designed to find drivers to access to medicines from a country’s health system perspective. This framework could enable the systematic evaluation of access across countries, disease areas and populations and facilitate targeted policy development. This framework is the byproduct of a larger study on the barriers and facilitators to childhood oncology medicines in South Africa.

**Results:**

Eight core (pharmaceutical) functional processes were identified from existing frameworks: (I) *medicine regulation*, (II) *public financing and pricing*, (III) *selection*, (IV) *reimbursement*, (V) *procurement and supply*, (VI) *healthcare delivery*, (VII) *dispensing* and (VIII) *use*. National contextual components included *policy and legislation* and *health information systems*. To emphasize the interlinkage of processes, the proposed framework was structured as a pharmaceutical value chain. This framework focusses on national processes that are within a country’s control as opposed to global factors, and functional mechanisms versus a country’s performance or policy objectives. Further refinement and validation of the framework following application in other contexts is encouraged.

## Introduction

Medicines are considered a key component of health systems and a major contributor to health outcomes [1]. Access to medicines is determined by the interaction between a multitude of factors and processes, not just in the pharmaceutical value chain but also within the broader context of the health system [2]. Research on accessibility of medicines is often focused on a particular process in isolation from related elements [3–5], or on the downstream effects for the patient– e.g. availability and affordability [6–10]. However, the entirety of the pharmaceutical system must be taken into consideration to get a comprehensive understanding of the drivers of accessibility.

To that end, we set out to get a better understanding of the drivers and barriers that determine access to pediatric oncology medicines in South Africa. To enable a comprehensive analysis of the issues influencing health system efficiencies and its ability to provide equitable access to childhood cancer medicines, we looked to available analytical tools to inform our qualitative analyses and development of an interview guide. The Paediatric Oncology System Integration Tool (POSIT) was reviewed and deemed suitable for constructing an interview guide [11]. POSIT was developed to facilitate analyses of the performance of childhood cancer programs in low- and middle-income countries (LMIC) within the context of their health system. Although medicines are only one element of a health system, many of the system functions and performance goals for health systems outlined in POSIT align with those for medicines.

However, thematic analysis of in-depth interviews with stakeholders soon revealed the limitations of POSIT in applying it specifically in the context of access to medicines, with several functional domains that are unique for pharmaceuticals missing from the framework. Missing elements included the regulation and registration of medicines, the selection of essential medicines, and preparing, dispensing and safe administration of pharmaceuticals. Other existing frameworks on access to medicines were either limited in scope or lacked specificity on pharmaceutical processes and were therefore not considered appropriate alternatives [2, 12–16].

With no single existing framework fit for the qualitative analysis of barriers and enablers in access to medicines, we aimed to develop a new analytical framework specifically designed to evaluate access to medicines from a health systems perspective within a country. Such a framework could yield a comprehensive health systems overview of drivers of access and concrete recommendations for improvement and policy development from stakeholders’ perspectives.

### Core components of an access to medicines framework

To inform core pharmaceutical functional processes, elements from two existing frameworks were used to construct a new framework: (1) the childhood cancer system functional domains in POSIT [11], and (2) the pharmaceutical management framework published in ‘MDS-3: Managing Access to Medicines and other Health Technologies’ [12]. *Managing Drug Supply-3* (MDS-3) is a reference guide detailing sustainable management of essential medicines in LMIC.

The analytical framework proposed by Bigdeli and colleagues was not used to construct our framework [2]. Although their framework is tailored to medicines and provides a complete overview of the complex components, levels and interconnections that determine access to medicines, it was developed for use in policy design whereas we sought to analyze drivers of access by understanding how effective collective action across the value chain through numerous stakeholders supports access to medicines. Additionally, we wanted to focus on national processes that are within a country’s sphere of influence as compared to global mechanisms. The framework proposed by the World Health Organization (WHO) in 2004 targets performance dimensions (e.g. *sustainability*, *affordability*, etc.) rather than functional processes (e.g. *regulation*, *reimbursement*, *dispensing*, etc.) and focusses on key outcomes for coordinated global action, and was therefore not used [13]. The WHO guidelines on developing National Medicines Policies [14] and derivatives [15, 16] are policy oriented and may not adequately capture the practical effects and lived experiences of such policies or the performance of functional pharmaceutical processes, and position medicines vertically rather than integrated in the health system.

**Fig. 1:**
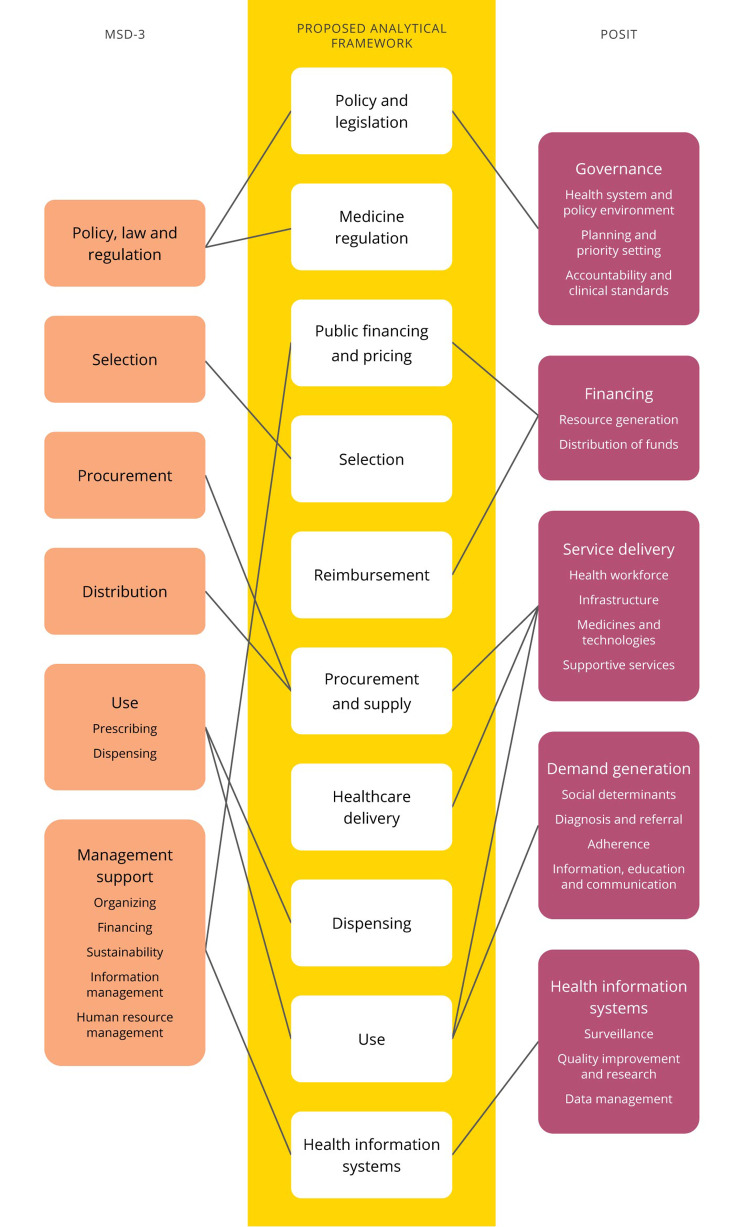
Mapping of POSIT [11] and MDS-3 [12] to construct a new analytical framework to identify barriers and facilitators to medicines’ access. Performance goals and dimensions as well as functional subdomains of POSIT not shown. POSIT: Paediatric Oncology System Integration Tool; MSD-3: Managing Drug Supply-3

The five functional domains of POSIT (e.g. *governance*, *financing*, *demand generation*, *health information systems* and *service delivery*) were combined with the four basic functions of pharmaceutical management (e.g. *selection*, *procurement*, *distribution* and *use* (including *prescribing* and *dispensing*)) and contextual elements *management support* and *policy, law and regulation*. Figure 1 provides a schematic representation of the generation of the framework.

**Fig. 2:**
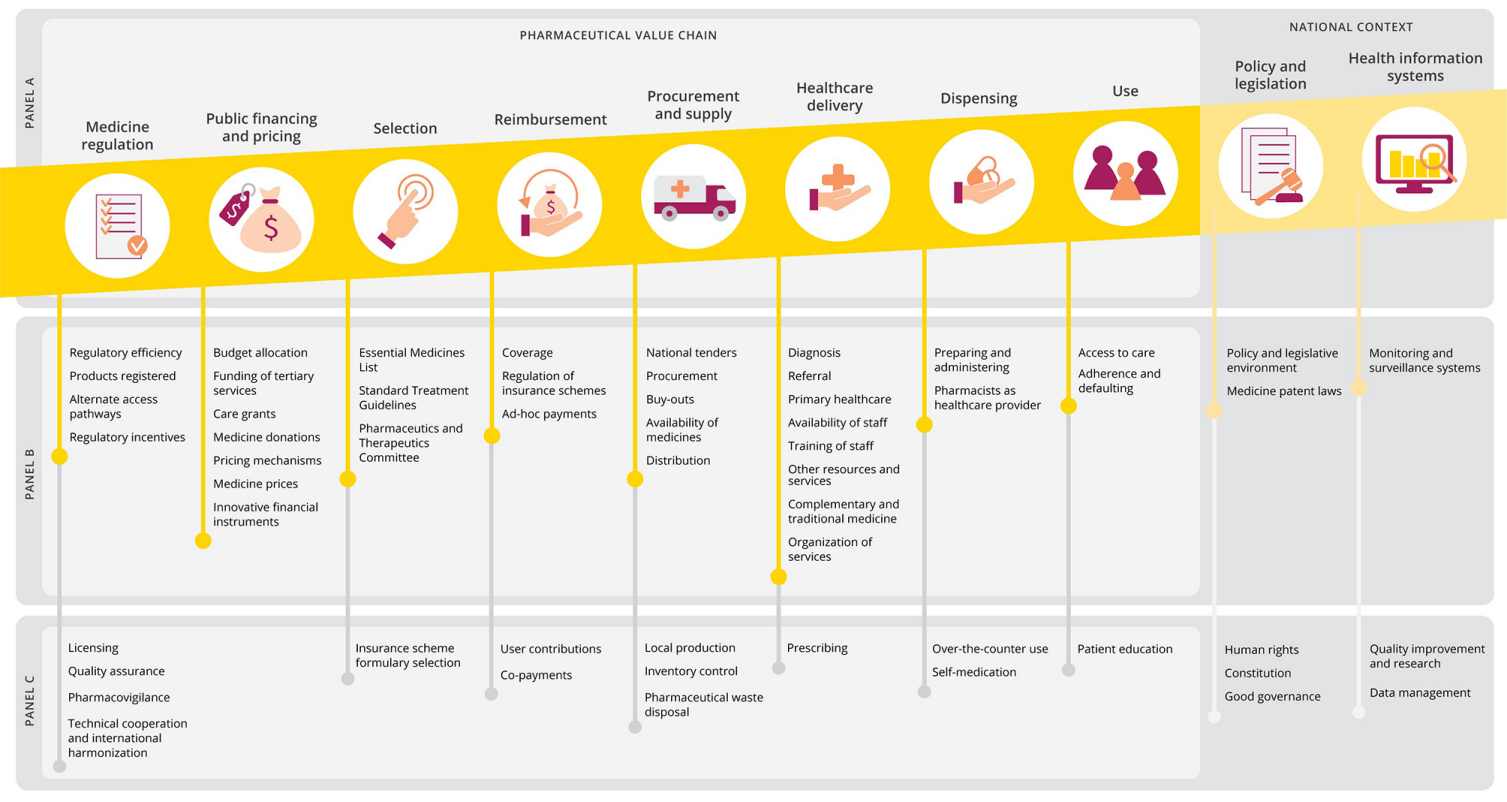
Proposed analytical framework for qualitative evaluation of barriers and facilitators in access to medicines. Panel A: general proposed analytical framework for access to medicines. Panel B: analytical framework applied to the qualitative study of barriers and facilitators in accessing childhood oncology medicines in South Africa (emerging cross-cutting themes not shown). Panel C: additional aspects to be considered in alternative contexts

From that, we identified eight core functional process: (I) *medicine regulation*, (II) *public financing and pricing*, (III) *selection*, (IV) *reimbursement*, (V) *procurement and supply*, (VI) *healthcare delivery*, (VII) *dispensing* and (VIII) *use* (including social and societal aspects). Other core components that influence the context under which the functional processes are taking place are *policy and legislation* and *health information systems*. Recognizing that each element builds on a previous component, we chose to map the framework as a pharmaceutical value chain (panel A, Fig. 2) [17]. This figure also illustrates how the framework was applied to the qualitative study of barriers and facilitators in access to pediatric cancer medicines in South Africa (panel B), while highlighting additional aspects that did not emerge in this specific case study but were recognized as potentially relevant elements in analogous frameworks (panel C) [11–16].

### Policy and legislation

*Policy and legislation* captures how the pharmaceutical system and broader healthcare structures within a country are organized, managed, and regulated through policies, laws or mandates [11]. The political environment is also covered within this theme.

### Medicine regulation

*Regulation* involves the marketing registration of medicines, pharmacovigilance activities, the licensing for manufacturing, distributing, storage and sale of pharmaceuticals, as well as importation and exportation [12]. Substandard and falsified medicines may also be considered here.

### Public financing and pricing

*Public financing* involves the generation, pooling, and allocation of public funds to cover medicines and services [11]. This may also include donations. Private funding is considered under *reimbursement*. *Pricing* considers the prices and affordability of medicines and mechanisms used to regulate prices. Frequently used pricing mechanisms include internal reference pricing, external reference pricing and value-based pricing [18].

### Selection

*Selection* encompasses the identification of prevalent health problems and selecting evidence based treatments of choice, choosing individual medicines and preferred dosage forms, and deciding which medicines will be available at each level of a health care system [12], usually in the form of a national Essential Medicines List (NEML) or formulary, and Standard Treatment Guidelines (STGs). This element also considers processes for making non- NEML or non-formulary listed medicines available to patients.

### Reimbursement

We consider *reimbursement* to include the coverage of pharmaceuticals in national or social medical insurance plans, subsequent reimbursement prices by third party payers, mechanisms to determine reimbursement prices, co-payments and the regulation of private sector medical insurance schemes [11, 19]. This element also considers processes for reimbursement/payment of medicines that are not covered by insurance schemes.

### Procurement and supply

*Procurement and supply* entails the selection and management of procurement methods– including tenders. In addition, distribution processes are also covered within this theme, encompassing aspects related to customs, stock control, and delivery to drug depots and health facilities [12]. Availability of medicines in health facilities is also considered here.

### Healthcare delivery

*Healthcare delivery* encompasses a range of structures, resources, services, healthcare professionals and other individuals required for the diagnosis and provision of care [11]. We consider *prescribing* of medicines to be part of this component.

### Dispensing

*Dispensing* comprises the process of preparing and giving or administering a medicine by a pharmacist or other healthcare professional to a named patient, frequently on the basis of a prescription [12]. However, over-the-counter (OTC) use and self-medication may also be considered here.

### Use

We consider *use* as the proper medicine consumption by the patient, as well as the ability of people to command appropriate healthcare resources. This includes patients’ knowledge on available health services and treatments, physical accessibility of services, and acceptability of services and medicines within associated social and societal structures [20].

### Health information systems

This component captures by which means data about disease burden and clinical patterns, health outcomes, and the achievement of objectives in the health system is collected, analyzed and reviewed [11, 12]. Monitoring and surveillance are a critical element herein.

## Discussion

In current literature, an increasing number of studies describe and analyze access to medicines from a country’s health system perspective [21–25], but a complete and suitable framework to facilitate a qualitative analysis was missing. In previous studies, elements from different frameworks needed to be combined to arrive at a suitable structure for analysis [22–25], similar to our own experience. This illustrates the necessity for an amended framework for qualitative research on access to medicines that encompasses the full scope of national functional domains in the pharmaceutical value chain. Similar to Bigdeli et al. and POSIT, we have adopted a health systems perspective on access to medicines, to highlight the interconnectedness of medicines and pharmaceutical processes with other key variables within the health system [2, 11].

Unlike most other existing framework, functional domains (‘*what we need to do’*) rather than performance dimensions (‘*what we aim to achieve’*) were taken as basis for this framework [2, 11, 13]. Although we consider these performance dimensions to be critical in policy design and development, the level of detail required to identify barriers is missing when performance is suboptimal. The proposed framework was specifically designed to address this gap in understanding how effective collective action across the value chain by numerous stakeholders supports access to medicines. Additionally, when applying our framework to a case study of childhood oncology medicines in South Africa, we have experienced that important performance dimensions of access spontaneously emerge (e.g. *availability*, *equity*, *affordability*, etc.), further enriching the findings.

Rather than providing a checklist through which one could perform a gap-analysis of whether specific policies or processes are in place, we provide an open structure for a qualitative assessment of how functional processes operate and how they impact access. For even when a given policy or process is theoretically in place, its practical effects and lived experiences may differ from what was intended. For example, a national tender process might be in place, but tenders can fail due to too strict participation requirements. Our open structure distinguishes our framework from prior works, and allows for more in-depth discussion and probing. Recognizing that existing frameworks and key documents were designed for different purposes, the proposed framework is meant to complement earlier work rather than replace them. An important strength of this novel framework is its intuitiveness. Critical processes that take place between the moment a medicine is registered for use in the country and actual use by the patient are compartmentalized to facilitate each component’s in-depth analysis, while also emphasizing the interaction between pharmaceutical processes, other healthcare services and actors and social factors. Correspondingly, all six WHO health system building blocks are captured within our framework [1]. The proposed framework was designed to be used in conjunction with different qualitative pharmaceutical policy analysis methods to derive a complete picture of the situation, including analyses of policy documents (such as a national medicines policy) and public information sources, key informant or patient interviews, and health facility surveys. Finally, in order to adequately capture all practical effects and lived experiences of existing policies and processes, analyses should encompass stakeholders across the value chain and not be restricted to policy-makers.

### Limitations

Inevitably, the compartmentalization of functional components in the pharmaceutical value chain oversimplifies the complexities of the health system and may underplay the importance of upstream factors that determine access to care. It also divides processes which are highly interlinked. At the same time, separating these components helps to put boundaries around complex processes, which minimizes the risk of key functional processes being overlooked, and thus facilitates identification of a range of barriers and enablers. Furthermore, this framework is not all-encompassing. We provide a general structure for systematic analysis of drivers of inaccessibility, but the analysis of access in different countries, therapeutic areas or populations may require the evaluation of other subdomains within these core components. We emphasize that this tool should not be considered a universal checklist, and adaptions to national health systems may be necessary. Additional cross-cutting themes that cannot be captured in a single core component may also be identified during analysis. Besides these emerging themes, global processes such as *market forces*, *innovation* and *manufacturing*– that undeniably have an impact on access to medicines as well– are not included in this framework as these are often beyond a country’s influence [2].

This framework is one outcome of a larger study looking into the barriers and facilitators to childhood oncology medicines in South Africa. With that, there was no protocolized, systematic approach to develop this framework. However, we have nonetheless taken careful approaches to ascertain that it reflects key processes and factors, having taken existing frameworks into consideration in the design of our framework [2, 11–13] and undertaken further verification through iterative discussions among authors.

## Conclusion

We propose a widely applicable analytical framework for studying qualitative access to medicines from a country’s health system perspective, outlining critical functional processes in the pharmaceutical value chain. We believe this framework could facilitate future analyses of barriers and enablers in accessing medicines, leading to a systematic understanding of determinants of access and potentially guiding targeted policy development. Although we expect the framework to be appropriate for studying other countries, diseases and populations in a structured manner, it is the derivative of a single case study in South Africa. It has yet to prove its usefulness across different contexts, and refinements may be needed to ensure its broad applicability and comprehensiveness. Testing and implementing the proposed framework in various contexts will contribute to its refinement and practical utility.

## Data Availability

Data sharing is not applicable to this article as no data from the study is described in the presentation of the proposed analytical framework.

## Abbreviations

LMIC: Low- and Middle-Income Countries
MDS-3: Managing Drug Supply edition 3
NEML: National Essential Medicines List
POSIT: Paediatric Oncology System Integration Tool
STG: Standard Treatment Guideline
WHO: World Health Organization
